# Health Care Costs Attributable to Prostate Cancer in British Columbia, Canada: A Population-Based Cohort Study

**DOI:** 10.3390/curroncol30030240

**Published:** 2023-03-08

**Authors:** Wei Zhang, Daphne P. Guh, Tima Mohammadi, Reka E. Pataky, Alexander C. T. Tam, Larry D. Lynd, Annalijn I. Conklin

**Affiliations:** 1 Centre for Health Evaluation and Outcome Sciences, Providence Research, St. Paul’s Hospital, 570-1081 Burrard Street, Vancouver, BC V6Z 1Y6, Canada; 2 School of Population and Public Health, University of British Columbia, 2206 East Mall, Vancouver, BC V6T 1Z3, Canada; 3 Canadian Centre for Applied Research in Cancer Control, BC Cancer, 675 West 10th Avenue, Vancouver, BC V5Z 1L3, Canada; 4 Faculty of Pharmaceutical Sciences, University of British Columbia, 2405 Wesbrook Mall, Vancouver, BC V6T 1Z3, Canada

**Keywords:** prostate cancer, healthcare costs, cost evaluation, administrative data

## Abstract

We aimed to estimate the total health care costs attributable to prostate cancer (PCa) during care phases by age, cancer stage, tumor grade, and primary treatment in the first year in British Columbia (BC), Canada. Using linked administrative health data, we followed a cohort of men aged ≥ 50 years at diagnosis with PCa between 2010 and 2017 (Cohort 1) from the diagnosis date until the date of death, the last date of observation, or 31 December 2019. Patients who died from PCa after 1 January 2010, were selected for Cohort 2. PCa attributable costs were estimated by comparing costs in patients to matched controls. Cohort 1 (*n* = 22,672) had a mean age of 69.9 years (SD = 8.9) and a median follow-up time of 5.2 years. Cohort 2 included 6942 patients. Mean PCa attributable costs were the highest during the first year after diagnosis ($14,307.9 [95% CI: $13,970.0, $14,645.8]) and the year before death ($9959.7 [$8738.8, $11,181.0]). Primary treatment with radiation therapy had significantly higher costs each year after diagnosis than a radical prostatectomy or other surgeries in advanced-stage PCa. Androgen deprivation therapy (and/or chemotherapy) had the highest cost for high-grade and early-stage cancer during the three years after diagnosis. No treatment group had the lowest cost. Updated cost estimates could inform economic evaluations and decision-making.

## 1. Introduction

Prostate cancer (PCa) is the most common cancer in men in Canada and many other countries and is the third leading cause of cancer-related death in men in Canada [1,2]. Unlike publicly funded screening programs for prevention or early detection of other common cancers such as lung cancer [3], colorectal cancer [4], cervical cancer [5], and breast cancer [6], the evidence supporting routine screening programs for PCa is mixed. On the one hand, the current prostate-specific antigen (PSA) testing has low specificity and leads to many false positive tests and thus unnecessary biopsies, overdiagnosis, and overtreatment [7,8,9]. Furthermore, the benefits of early active treatment are not clear. For example, Bill-Axelson and colleagues found survival benefits in radical prostatectomy (RP) compared to watchful waiting (WW) among men with localized PCa at a 29-year follow-up [10]. However, other randomized clinical trials with relatively shorter follow-up, such as the ProtecT trial and PIVOT, did not show RP was superior to WW [11,12,13]. 

Economic evaluations have been conducted to evaluate the cost-effectiveness of different screening programs, new and more accurate screening tests, or treatment strategies for PCa [9,14,15,16,17,18]. Updated cost estimates are important model inputs in the decision-analytic models commonly used in these economic evaluations, which are widely required for evidence-based funding decisions by public or private health insurance programs. Therefore, the cost estimates inform not only the burden of illness but also the economic evaluations.

Most of the PCa costing studies before 2010 used one-year data and reported annual costs or first-year costs after diagnosis, and only a few studies used longitudinal data and estimated the costs over multiple years [19,20]. In recent years, there have been increasing needs and trends to use large administrative health data sets to construct a cohort of people with a specific health condition and follow them longitudinally to capture real-world health care utilization and costs. For PCa, recent costing studies based on such data and design have measured and compared the costs by factors such as age, disease state or stage, tumor grade, treatment strategy, and/or care phase (including the diagnosis phase before diagnosis) [21,22,23,24,25]. However, previous data were either relatively outdated and thus not well representative of the most recent PCa treatment guidelines or only measured costs by some factors instead of all. The objective of our study was to estimate the direct health care costs attributable to PCa over different care phases, by age, PCa stage, tumor grade, and treatment, using population-based cohorts constructed from the administrative health data in British Columbia (BC), Canada. 

## 2. Materials & Methods

### 2.1. Data Source, Patient Cohorts, and Matched Controls

We used the administrative health data from BC Cancer and Population Data BC [26,27,28,29,30,31,32,33] for men aged ≥ 50 years old who were BC residents and registered for BC’s public health insurance program between 1 January 2010 and 31 December 2019. The data included: (1) BC Cancer data for information on cancer diagnosis, cancer stage and tumor grade at diagnosis, and cancer treatment, consultation and follow up [26,27]; (2) Medical Services Plan (MSP) for information on physician services and billings under BC’s public health insurance program [28]; (3) PharmaNet for information on all prescription drugs and medical supplies dispensed from community pharmacies and hospital outpatient pharmacies for patient use at home and the corresponding costs regardless of payer [29]; (4) Discharge Abstracts Database (DAD) for information on hospitalization and costs [30]; (5) National Ambulatory Care Reporting System (NACRS) for information on emergency department (ED) visits and costs [31]; (6) BC Vital Events and Statistics Deaths [32]; and, (7) Central Demographics File for information on demographics (age and sex), MSP registration, health service delivery area, and neighborhood income [33]. 

We constructed a cohort of men aged ≥ 50 years old at diagnosis with PCa between 1 January 2010 and 31 December 2017 (Patient Cohort 1) and followed them from six months before the date of diagnosis until the date of death, the last date of observation, or 31 December 2019, whichever came first. The time period was selected because PCa cancer stage and Gleason score at diagnosis were only captured in BC Cancer data from 2010 to 2017. Patients who were diagnosed with PCa after 1 January 1997 and died from PCa after 1 January 2010 were selected to form Patient Cohort 2. The observation period included 5 intervals: (I) before diagnosis (6 months before diagnosis); (II) initial care (12 months after diagnosis based on Cohort 1); (III) post-initial care (12–24 months after diagnosis based on Cohort 1); (IV) continuing care (24 months after diagnosis till the earliest of 12 months before death, the last date of observation, or 31 December 2019 based on Cohort 1); and (V) terminal care (12 months before death based on Cohort 2). The intervals were determined based on previous studies [20,24,34], and confirmed by plotting the cost per year since diagnosis (Supplementary Figure S1).

Up to 2 controls for the cases in each cohort were matched from the BC general population not diagnosed with PCa by age (within 2 years), health service delivery area (16 areas covering the province of BC used to provide program delivery and services), neighborhood income quintile (Quintile of Annual Income Per Person Equivalent), Elixhauser Comorbidity Index [35] (0, 1, 2, 3+), and the year of last follow-up. Age, health service delivery area, and neighborhood income quintile were evaluated at the time of diagnosis, whereas the Elixhauser comorbidity index was derived using the last 2 years of DAD data prior to PCa diagnosis. Patients in Cohort 2, who died from PCa, were matched to controls, who died from other causes. The study was approved by the behavioral research ethics board of the University of British Columbia (H20-01258).

### 2.2. Outcome Measures

Total health care costs included costs for physician services, prescription medications, hospitalizations, ED visits, and PCa treatments. Physician service costs were estimated from physician billing records in the MSP database, which contained medically necessary services provided by fee-for-service practitioners and some shadow billings for non-fee-for-services in BC. Prescription medication costs, including ingredient costs, professional fees, and special service fees, were estimated through pharmacy dispensing records in the PharmaNet database. Hospitalization costs, including day procedure costs, were estimated by multiplying the case mix resource intensity weight reported in DAD by the cost of a standard hospital stay in BC, as reported by the Canadian Institute for Health Information [36]. The costs of ED visits were estimated by multiplying the number of unique ED visits identified from NACRS, MSP, and DAD [37] by the BC facility cost per visit [38]. 

PCa treatment costs only applied to patient cohorts. Cancer patients in BC received care through BC Cancer (BC Cancer Regional Centre or Community Oncology Network clinic). We used the BC Cancer database and the corresponding unit cost to estimate PCa treatment costs, including the costs of radiation therapy (RT) [39] (i.e., beam radiation, brachytherapy, radiation planning), medication costs of androgen deprivation therapy (ADT), and chemotherapy that were approved by BC Cancer for PCa and dispensed from the BC Cancer Pharmacy [40,41,42], as well as oncologists’ consultations and follow-ups using their midpoint salary rate [43]. The costs of surgical treatments, including RP and other PCa-related surgeries, were estimated using DAD. 

### 2.3. Grouping by Cancer Stage, Tumor Grade, and Treatment 

Cancer staging was based on how much cancer was in the body at diagnosis, whether it had spread, and where it had spread to. We categorized PCa into early (stages T1 and T2) vs. advanced (stages T3 and T4) stages and low grade (Gleason score ≤ 6) vs. high grade (Gleason score > 6). Primary treatment was determined by the treatments received in the first year after the diagnosis in the order of: (1) RP or other PCa-related surgeries [44]; (2) RT; (3) ADT (and/or chemotherapy); and (4) no treatment (i.e., WW or active surveillance (AS) and not receiving any of the above treatments). Supplementary Table S1 lists all treatments considered.

### 2.4. Statistical Analysis

Costs attributable to PCa were estimated by comparing costs in cases in both patient cohorts to their matched controls from the general population of men aged ≥ 50 years. The total cost observed for each individual in each interval was divided by the individual’s follow-up time in the interval and then standardized to the cost per 100 days. For each interval, we estimated the mean cost per 100 days for the cases and controls (and then the mean cost attributable to PCa) using a generalized estimating equation (GEE) linear regression model where the case-control matches were considered clusters. The total cost per interval was then calculated by multiplying the estimated cost per 100 days by the length of the interval. As a sensitivity analysis, we also used a GEE generalized linear model (GLM) with gamma distribution. 

Attributable costs were further stratified by age (<65 years vs. ≥65 years) [10,11,12,13], cancer stage, tumor grade at diagnosis, and primary treatment. Respective costs were estimated by fitting similar GEE regression models separately by age. In addition to the entire Cohort 1, we performed a subgroup analysis of those patients who had at least 5 years of follow-up (i.e., survived at least 5 years since diagnosis). Furthermore, we conducted a sensitivity analysis by classifying a Gleason score ≤ 3 + 4 as low grade versus a Gleason score ≥ 4 + 3 as high grade.

All costs were inflated to 2021 Canadian dollars ($) using the Consumer Price Index for health and personal care reported by Statistics Canada [45]. There were no missing data on health care service utilization and costs, but there were missing data on cancer stage and tumor grade. All the analyses by cancer stage or tumor grade were conducted among those without missing data. All statistical analyses were performed using SAS Version 9.4 and R Version 4.0.5.

## 3. Results

**Figure 1:**
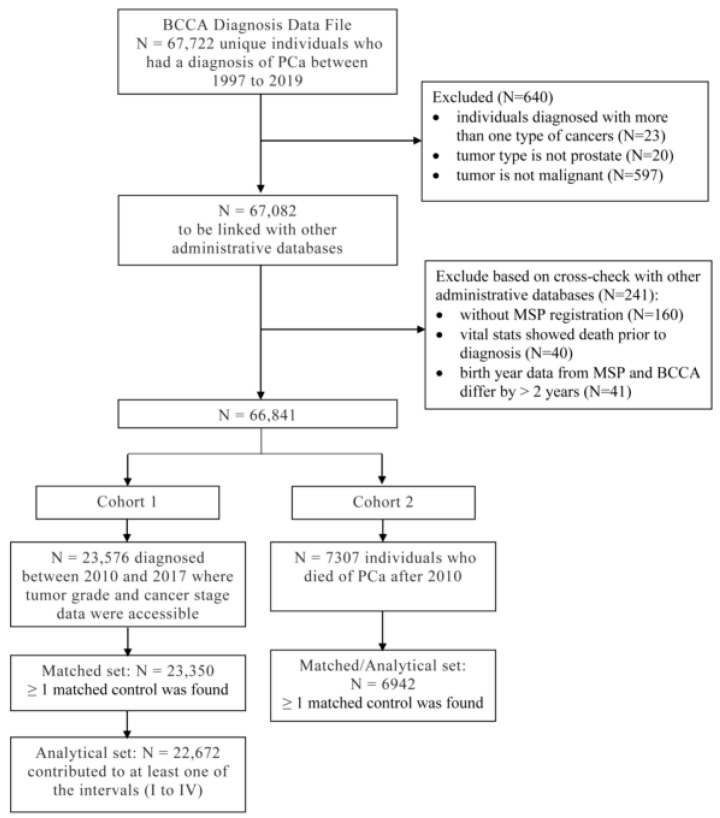
Cohort selection flowchart. BCCA: British Columbia Cancer Agency; PCa: prostate cancer; MSP: Medical Services Plan.

Figure 1 presents the selection process for each patient cohort and their matched cohort. Patient Cohort 1 included 22,672 PCa patients, and their matched control cohort included 45,420 controls. Patient Cohort 2 and their matched cohort included 6942 patients and 13,427 controls, respectively. 

The cases and controls were comparable in terms of age, health service delivery area, neighborhood income quintile, Elixhauser Comorbidity Index, and follow-up time because the differences were not statistically significant (Supplementary Table S2). The average age was 69.9 (SD = 8.9), with 71.3% of the population aged ≥ 65 years in Cohort 1. The majority of the patients were diagnosed at an early stage (63.6%) and at a high grade (70.9%). The distribution of primary treatment was 31.4% receiving RP or other PCa-related surgeries, 27.5% receiving RT, 15.3% receiving ADT/chemotherapy, and 25.8% receiving no treatment. The median follow-up time was 5.2 [IQR: 3.1–7.5] years. The patients in cohort 2 were older than those in cohort 1, with a mean age at diagnosis of 75.1 (SD = 9.5) years. Patient characteristics were different by age group in Cohort 1 (Table 1). Patients aged ≥ 65 years had shorter follow-up times and were more likely to be at an advanced stage, have a high grade, and be treated with ADT/chemotherapy in the first year than patients aged < 65 years.

### 3.1. Costs by Intervals 

The number of individuals and their follow-up duration considered in each interval were presented in Supplementary Table S3. The costs per 100 days in cases were higher than controls in all intervals (Supplementary Tables S3 and S4). The mean attributable costs (95% CI) per 100 days estimated using GEE linear regression (similar to the results using GEE GLM with gamma distribution) were the lowest before diagnosis (Interval I: $396.4 ($334.6 to $458.1)), the highest during initial care after diagnosis (Interval II: $3920.0 ($3827.4 to $4012.5)), and then the terminal care before death (Interval V: $2728.7 ($2394.2 to $3063.3)) (Supplementary Table S3). The mean attributable costs (95% CI) per 100 days post-initial care (Interval III) were similar to those during continuing care (Interval IV). Based on the estimated costs per 100 days, the total attributable costs were $14,307.9 ($13,970.0 to $14,645.8) during the year after diagnosis and $9959.7 ($8738.8 to $11,181.0) during the year before death, respectively. 

### 3.2. Costs by Age, Cancer Stage, and Tumor Grade 

**Figure 2:**
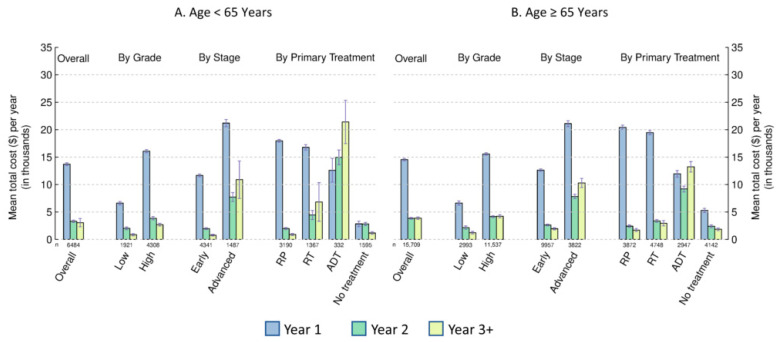
Estimated mean total costs attributable to prostate cancer per year after diagnosis by age, cancer stage, tumor grade, and primary treatment in the first year. Panel (**A**): patients under age 65 years; Panel (**B**): patients 65 years and older; Year 1 or Interval II: initial care (12 months after diagnosis based on Cohort 1); Year 2 or Interval III: post-initial care (12–24 months after diagnosis based on Cohort 1); Year 3+ or Interval IV: continuing care (24 months after diagnosis till the earliest of the last 12 months before death, the last date of observation, or 31 December 2019 based on Cohort 1). Grey bars are standard errors. RP = radical prostatectomy or other PCa-related surgeries; RT = radiation therapy; ADT = androgen deprivation therapy and/or chemotherapy.

For each patient subgroup (age, stage, and grade), the mean total attributable cost per year showed a sharp decline after the first year of initial care (Figure 2A,B, based on Cohort 1; Supplementary Table S5). The mean cost per year in the second year post-initial care was generally higher than that in continuing care among all subgroups by age, stage, and grade, except for the advanced stage groups and the group aged ≥ 65 years with a high grade. The mean total cost per year for high grade or advanced stage was higher than that for low grade or early stage in both age groups, respectively. For both age groups, the estimated mean cost before diagnosis (Interval I) for low grade tended to be higher than the estimated mean cost in Year 3+ (Interval IV) (Supplementary Tables S4 and S5). 

### 3.3. Costs by Primary Treatment

**Figure 3:**
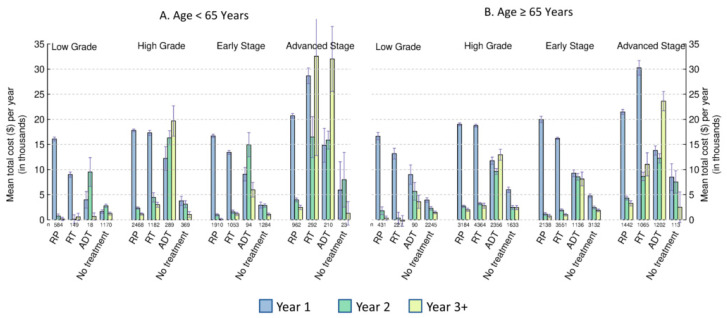
Estimated mean total costs attributable to prostate cancer per year after diagnosis by primary treatment in the first year. Panel (**A**): patients under age 65 years; Panel (**B**): patients 65 years and older; Year 1 or Interval II: initial care (12 months after diagnosis based on Cohort 1); Year 2 or Interval III: post-initial care (12–24 months after diagnosis based on Cohort 1); Year 3+ or Interval IV: continuing care (24 months after diagnosis till the earliest of the 12 months before death, the last date of observation, or 31 December 2019 based on Cohort 1). Grey bars are standard errors. RP = radical prostatectomy or other PCa-related surgeries; RT = radiation therapy; ADT = androgen deprivation therapy and/or chemotherapy.

The mean total attributable costs per year in the three intervals after diagnosis (year 1, year 2, and year 3+) for RT, ADT/chemotherapy, and no treatment showed different patterns between the two age groups (Figure 2A,B). Within each age group, the first-year costs were the highest for low-grade or early-stage patients receiving RP/surgery as their primary first-year treatment, followed by costs for RT, ADT/chemotherapy, and no treatment (Figure 3A,B, based on Cohort 1). The costs in the second and third years and beyond were the highest for men receiving ADT/chemotherapy. Thus, total costs of ADT/chemotherapy during the three years after diagnosis were the highest in early-stage patients (Supplementary Figure S2). 

The cost patterns over the three intervals for high grade were similar between the two age groups, except for ADT/chemotherapy treatment (Figure 3A,B). The costs for high-grade patients aged < 65 years receiving ADT/chemotherapy increased across years, and their costs in years 2 and 3+ were much higher than their counterparts aged ≥ 65 years. ADT/chemotherapy had the highest cost after the first year, which led to the highest cumulative total costs during the three years after diagnosis among the four treatment groups (Supplementary Figure S2). 

In patients with an advanced stage, RT had the highest first-year cost among treatment groups and higher costs in years 2 and 3+ than the RP/surgery treatment group. ADT/chemotherapy had similar costs to RT after the first year in the age group < 65 years but the highest cost after the first year in the age group ≥ 65 years. Thus, total costs in the three years after diagnosis were much higher for RT and ADT/chemotherapy than for RP and no treatment.

When comparing the estimated mean cost in Intervals I–IV by primary treatment, we found that the estimated mean cost before diagnosis (Interval I) for the no treatment group tended to be higher than their estimated mean cost in Year 3+ (Interval IV) and was the highest among the four primary treatment groups (Supplementary Tables S4 and S5).

### 3.4. Subgroup and Sensitivity Analysis

The patient characteristics of the subgroup who had at least 5 years of follow-up were shown in Supplementary Table S6, and their estimated costs were presented in Supplementary Figure S3. In those aged ≥ 65 years, the subgroup patients were slightly younger compared to the entire Cohort 1. In both age groups, the subgroup patients were more likely to be at an early stage, of low grade, and treated with RP or other PCa-related surgeries. The estimated mean total attributable costs per year among the subgroup at an advanced stage or primarily treated with ADT/chemotherapy were lower, especially in Years 3+, than those in the entire Cohort 1.

We applied an alternative categorization of low-grade versus high-grade as sensitivity analysis. The key difference between the sensitivity analysis and the main analysis was to split those with a total Gleason score = 7 (classified as high grade in the main analysis) into low grade (Gleason score = 3 + 4) or high grade (Gleason score = 4 + 3) based on Gleason’s primary pattern and secondary pattern values. Of those with a Gleason score = 7 (*N* = 9754), 6022 (61.7%) were 3 + 4, 3678 (37.7%) were 4 + 3, and 54 (0.6%) had missing data on Gleason’s primary pattern and secondary pattern values. Supplementary Table S7 presents the patient characteristics based on the alternative categorization. Supplementary Figures S4 and S5 present the estimated mean costs attributable to PCa after diagnosis for both categorizations. The mean costs shown in Supplementary Figures S4 and S5 for the alternative categorization can also be found in Supplementary Table S8. As expected, the mean cost in Year 1 for low-grade items based on the alternative categorization was higher. Patients with missing Gleason’s primary and secondary pattern values (*N* = 54) were excluded from this sensitivity analysis. These excluded patients had a lower mean cost in the first year, and thus, not including them led the mean cost for high-grade patients based on the alternative categorization to be higher.

## 4. Discussion

Using administrative health data from BC Cancer and Population Data BC in 2010–2019, we estimated the direct health care costs attributable to PCa over five different care phases by comparing the costs in PCa patients with their matched controls from the BC general population not diagnosed with PCa. We found that initial care (12 months after diagnosis) cost the most, followed by terminal care (12 months before death). We further estimated the PCa attributable costs in the years after diagnosis by stratifying cases and controls by age, cancer stage, tumor grade, and primary treatment received in the first year after diagnosis. The mean attributable cost per year was higher for high grades or advanced stages than the cost per year for low grades or early stages in both age groups. The attributable costs per year for four primary treatments varied by age, grade, and stage, except for the no treatment group, where they consistently cost the least during the three years after diagnosis. 

A few cost-effective studies using administrative data classify PCa patients by age, stage, tumor grade, and treatment [20,21,22,23,24,46]. We limit our discussion to similar studies conducted in Canada or the most recent studies using data covering 2010 or later years in other western countries, where clinical guidance may be closer to Canada, for better comparisons with our study. The three Canadian studies using the data from the province of Ontario also divided observation time into different care phases and found that the costs for PCa patients were the highest during the early stage of care and at the end of life [20,21,46]. However, they found the costs during the terminal care phase were higher than the costs in the initial care phase, which is opposite to our finding. One possible explanation is that two of the studies [20,21] distinguish pre-terminal care (18 months to 6 months before death) from terminal care (6 months before death), whereas our study only defined terminal care as 12 months before death. Another possible reason is that we did not consider the costs of long-term care and home care, which are usually higher for end-of-life care [46]. In our study, no treatment had the lowest costs for the initial care interval and over subsequent periods after diagnosis, which was consistent with two recent studies from the US [22,24]. However, results for RT contrasted. Magnani et al. (2021) found that RT cost more than surgery for both favorable and unfavorable-risk PCa in a cohort of men aged 35 to 89 years in the California Cancer Registry [22]. Using Medicare data, Tang et al. (2020) found the costs in the first two years were the highest among patients aged >65 years receiving stereotactic body radiation therapy, followed by RP, brachytherapy, and AS [23]. Our study found that RT costs more than RP/surgery only for advanced stages, and we also did not estimate brachytherapy costs separately from RT costs.

Our study has several limitations. First, unit costs for ED visits, RT, ADT, and chemotherapy were obtained from external sources [38,39,40,41,42,43]. However, these external sources were reliable and commonly used for costing purposes, and costs were directly available for all other categories. Second, approximately 30% of physicians in BC receive remuneration through alternative payment plans, i.e., service contracted and salaried physicians [47], and about 20% of total physician payments in BC were through alternative payment plans [48]. Thus, the MSP database captures most but not all of these shadow billings. Therefore, the physician costs for both cases and controls were likely to be underestimated. The cost differences between cases and controls could be underestimated as well, as the same study found that >20% of medical specialist payments were non-MSP [48]. Third, we did not consider the costs of home and community care because of the low data quality [49]. The exclusion of these costs might impact the cost estimates for terminal care duration because of the expected higher home and community care services at the end of life. In addition, WW and AS are different in practice, but we had to group them together as one treatment group because of the challenges of costing AS separately. While some local guidance is in place for BC for men with low-risk PCa, uptake of AS is not uniform in Canada, and its identification requires careful chart abstraction [50]. As part of AS, patients could be monitored by imaging, repeat biopsies, etc., which was not fully reflected in our data, and so these costs are likely an underestimate. Further, we defined primary treatment groups based on the treatment received in the first year after diagnosis. Patients may receive other treatments later; for example, no treatment group (WW/AS) may seek curative treatment later. Although the treatment received later was not reclassified or could be censored due to loss to follow-up, its costs were reflected in the figure for the later phases. 

Furthermore, we had data on cancer staging and tumor grade at diagnosis; however, we did not have data on cancer progression (and we did not have long enough follow-up data on those who were more recently diagnosed) to allow us to examine the disease trajectory (or state) as well as the cost of care trajectory (or state) over the lifetime. Thus, although we found the cost in the year before death was lower than the cost in the initial care phase, it is possible that in certain years over the disease trajectory (e.g., metastatic castration-resistant PCa), the health care costs could be much higher than those in the initial care phase, which could not be fully captured using our data. However, in our study, we did find that the mean cost per year in years 3 and beyond in the ADT/chemotherapy primary treatment group was higher than its cost in year 1 among patients with high grades or in advanced stages.

The strengths of this study include the fact that we used population data with detailed and comprehensive health care utilization and claims records from the BC health system perspective as well as distinguished the observation time into five distinct intervals. A strength of our data is that we could further estimate costs by cancer stage, tumor grade, and primary treatment. We selected matched controls from the BC general population without PCa for our patient cohorts to estimate the incremental costs among patients with PCa (i.e., costs attributable to PCa) by assuming the two populations have comparable non-PCa-related health care costs. This enabled us to better capture costs related to mild symptoms before PCa diagnosis and adverse events or complications from PCa treatments. Another strength is that we used the most recent data from 2010–2019 with a relatively longer follow-up duration (median follow-up of 5.2 years) than in previous studies [20,21,22,23,24]. 

Our study findings better reflect the recent PCa treatment practice in BC and could have implications for some Western countries with comparable clinical practices. For example, the high second and third year (and beyond) costs associated with ADT/chemotherapy among those with high-grade or advanced-stage PCa in men aged < 65 years may be attributable to concurrent therapy options such as initiation on neoadjuvant deprivation therapy for eight months before it is used as an adjuvant therapy to RT for up to three years [51]—resulting in higher costs beyond the first year. Higher costs in year 3+ may also reflect the need for secondary interventions to manage toxicities associated with androgen suppression [52,53]. Our approach of estimating treatment costs by a multitude of factors reflects the complex risk-based approach to PCa management that is recommended in both local guidance [51] and guidance in other western jurisdictions [54,55]. The primary treatment received by PCa patients might have been determined by clinicians and patients’ preferences and informed by prior effectiveness and cost-effectiveness evidence. The primary treatment received by PCa patients would then affect their clinical outcomes, health care costs, and quality and quantity of life. The cost-effectiveness analysis of a new intervention takes account of both costs and quality-adjusted life years (QALYs), a measure combining the quality and quantity of life. The outcome of cost-effectiveness analysis is often presented as the incremental cost-effectiveness ratio, i.e., the cost difference between the new intervention and the current practice divided by their QALY difference. Our cost estimates can be used in future cost-effectiveness analyses examining whether a new screening/diagnostic technology for PCa or a new PCa treatment is cost-effective. 

## 5. Conclusions

The costs attributable to PCa were the highest in the first year after diagnosis and at the end of life, and they also varied by age, cancer stage, tumor grade, and primary treatment. The attributable costs among patients at an early stage of cancer or low grade were much lower than those of other patients. RP/surgery cost significantly more than RT during the first year after diagnosis, but cost similarly after the first year for low-grade and early-stage cancer. RT costs are much higher each year after diagnosis than RP/surgery for advanced stages. ADT/chemotherapy may be the most expensive treatment strategy for advanced-stage patients over the age of 65, as well as for early-stage and high-grade patients. The attributable costs of patients with no treatment remained the lowest, which suggested the potential cost savings from minimizing overdiagnosis and overtreatment of PCA. This study could inform the burden of illness as well as model input parameters for economic evaluations to aid future healthcare decision-making. 

## Figures and Tables

**Table 1:** Characteristics of patients with prostate cancer at diagnosis (Cohort 1) by age group.

Variable	Age < 65 (*N* = 6518)	Age ≥ 65 (*N* = 16,154)
Age at diagnosis		
Mean (SD)	59.5 (3.64)	74.1 (6.75)
Median (IQR)	60.0 (57.0, 63.0)	73.0 (69.0, 79.0)
Follow-up in years		
Mean (SD)	5.9 (2.46)	5.1 (2.54)
Median (IQR)	6.1 (3.7, 8.1)	4.8 (2.9, 7.2)
PCa Stage, *n* (%)		
Early	4354 (66.8%)	10,073 (62.4%)
Advanced	1507 (23.1%)	4059 (25.1%)
Missing	657 (10.1%)	2022 (12.5%)
Tumor Grade, *n* (%)		
Low grade	1924 (29.5%)	3022 (18.7%)
High grade	4325 (66.4%)	11,744 (72.7%)
Missing	269 (4.1%)	1388 (8.6%)
Stage and Grade, *n* (%)		
Early, low grade	1536 (23.6%)	2386 (14.8%)
Early, high grade	2673 (41.0%)	7333 (45.4%)
Advanced, low grade	77 (1.2%)	106 (0.7%)
Advanced, high grade	1344 (20.6%)	3304 (20.5%)
Missing	888 (13.6%)	3025 (18.7%)
Primary Treatment, *n* (%)		
RP or other PCa-related surgeries	3198 (49.1%)	3920 (24.3%)
RT	1382 (21.2%)	4858 (30.1%)
ADT and/or chemotherapy	338 (5.2%)	3126 (19.4%)
No treatment	1600 (24.5%)	4250 (26.3%)
Early stage: Primary Treatment, *n* (%)		
RP or other PCa-related surgeries	1916 (44.0%)	2169 (21.5%)
RT	1054 (24.2%)	3562 (35.4%)
ADT and/or chemotherapy	96 (2.2%)	1164 (11.6%)
No treatment	1288 (29.6%)	3178 (31.5%)
Advanced Stage: Primary Treatment, *n* (%)		
RP or other PCa-related surgeries	964 (64.0%)	1457 (35.9%)
RT	306 (20.3%)	1162 (28.6%)
ADT and/or chemotherapy	213 (14.1%)	1309 (32.2%)
No treatment	24 (1.6%)	131 (3.2%)
Low grade: Primary Treatment, *n* (%)		
RP or other PCa-related surgeries	585 (30.4%)	434 (14.4%)
RT	149 (7.7%)	228 (7.5%)
ADT and/or chemotherapy	18 (0.9%)	91 (3.0%)
No treatment	1172 (60.9%)	2269 (75.1%)
High grade: Primary Treatment, *n* (%)		
RP or other PCa-related surgeries	2469 (57.1%)	3196 (27.2%)
RT	1192 (27.6%)	4426 (37.7%)
ADT and/or chemotherapy	294 (6.8%)	2454 (20.9%)
No treatment	370 (8.6%)	1668 (14.2%)

SD: standard deviation; IQR: interquartile range (1st quartile, 3rd quartile); PCa: prostate cancer; RP: radical prostatectomy; RT: radiation therapy; ADT: androgen deprivation therapy.

## Data Availability

The data that supports the findings of this study are available from Population Data BC (https://www.popdata.bc.ca/, accessed on 28 September 2021), but restrictions apply to the availability of these data. The data were used under approval and research agreements with the Data Stewards, and so are not publicly available. Access to the data provided by the Data Steward(s) is subject to approval but can be requested for research projects through the Data Steward(s) or their designated service providers.

## Supplementary Materials

The following supporting information can be downloaded at: https://www.mdpi.com/article/10.3390/curroncol30030240/s1. Table S1: List of treatments; Table S2: Matched characteristics of patients with prostate cancer (cases) and controls at diagnosis (Cohort 1) and at 12 months before death (Cohort 2); Table S3: Duration of follow-up and costs per 100 days for cases and controls by interval; Table S4: Costs per 100 days for cases in Cohort 1 and controls by cancer stage, tumor grade, and primary treatment; Table S5: Estimated mean total costs attributable to prostate cancer six months prior to diagnosis and per year after diagnosis by age, cancer stage, tumor grade, and primary treatment in the first year; Table S6. Subgroup analysis: Characteristics of patients with prostate cancer at diagnosis (those with at least 5 years of follow-up) by age group; Table S7. Sensitivity Analysis: Comparison of Patient Characteristics Based on Two Definitions of Tumor Grade; Table S8. Sensitivity analysis: Estimated mean total costs attributable to prostate cancer six months prior to diagnosis and per year after diagnosis by age, tumor grade (Gleason ≤ 3 + 4: low grade and Gleason ≥ 4 + 3: high grade), and primary treatment in the first year; Figure S1: Observed mean cost per 100 days for cases and controls in Cohort 1 by year; Figure S2: Estimated mean total costs in the first three years from diagnosis by age, cancer stage, tumor grade, and primary treatment; Figure S3. Subgroup analysis: estimated mean total costs attributable to prostate cancer per year after diagnosis by age, cancer stage, tumor grade, and primary treatment in the first year among those with at least 5 years of follow-up; Figure S4. Sensitivity analysis: estimated mean total costs attributable to prostate cancer per year after diagnosis by age and two definitions of tumor grade (Figure S5). Sensitivity analysis: estimated mean total costs attributable to prostate cancer per year after diagnosis by age, primary treatment in the first year, and two definitions of tumor grade.
