# Supplementary material for: Health Care Costs Attributable to Prostate Cancer in British Columbia, Canada: A Population-Based Cohort Study
Source: Curr Oncol. 2023 Mar 8;30(3):3176–88. doi: 10.3390/curroncol30030240 (PMC10047657; doi:10.3390/curroncol30030240)
Supplement: Supplementary file 1 [file curroncol-30-00240-s001.zip › curroncol-2244892-supplementary.pdf]

Online Supplementary Materials

Table S1. List of treatments 2

Table S2. Matched characteristics of patients with prostate cancer (cases) and controls at diagnosis (Cohort 1) and at 12 months before death (Cohort 2) 6

Table S3. Duration of follow-up and costs per 100 days for cases and controls by interval 10

Table S4. Costs per 100 days for cases in Cohort 1 and controls by cancer stage, tumor grade and primary treatment 12

Table S5. Estimated mean total costs attributable to prostate cancer six months prior diagnosis and per year after diagnosis by age, cancer stage, tumor grade and primary treatment in the first year 16

Table S6. Subgroup analysis: Characteristics of patients with prostate cancer at diagnosis (those with at least 5 years of follow-up) by age group 21

Table S7. Sensitivity analysis: Comparison of patient characteristics based on two definition of tumor grade 23

Table S8. Sensitivity analysis: Estimated mean total costs attributable to prostate cancer six months prior diagnosis and per year after diagnosis by age, tumor grade (Gleason ⩽3+4: low grade and Gleason ⩾4+3: high grade) and primary treatment in the first year 24

Figure S1. Observed mean cost per 100 days for cases and controls in Cohort 1 by year 26

Figure S2. Estimated mean total costs in the first three years from diagnosis by age, cancer stage, tumor grade and primary treatment 27

Figure S3. Subgroup analysis: Estimated mean total costs attributable to prostate cancer per year after diagnosis by age, cancer stage, tumor grade and primary treatment in the first year among those with at least 5 years of follow-up 28

Figure S4. Sensitivity analysis: Estimated mean total costs attributable to prostate cancer per year after diagnosis by age, and two definitions of tumor grade 29

Figure S5. Sensitivity analysis: Estimated mean total costs attributable to prostate cancer per year after diagnosis by age, primary treatment in the first year and two definitions of tumor grade 30

Table S1. List of treatments

**Primary Treatment Group**	**Data Source**	**Code**[a]	**Treatment**	**Approved Indication**
Radical prostatectomy or other prostate cancer related surgeries[b]	DAD	1.QT.91.^^	Excision radical, prostate	
		1.QT.59.^^	Destruction, prostate	
		1.PM.91.^^	Excision radical, bladder	
		1.PM.92.^^	Excision radical with reconstruction, bladder NEC	
		1.QM.89.^^	Excision total, testis	
		1.QM.91.^^	Excision radical, testis	
		1.MH.87.^^	Excision partial, lymph node(s), pelvic	
		1.MH.89.^^	Excision total, lymph node(s), pelvic	
Radiation therapy[c]	BCCA radiation treatment database	R	Radiotherapy	
		B	Brachytherapy	

Androgen deprivation therapy and/or chemotherapy[d]	BCCA pharmacy data with dispensation records of any of these drugs that were approved for Prostate Cancer by BCCA	GUPCABA	cabazitaxel	palliative therapy for metastatic castration resistant prostate cancer using cabazitaxel and prednisone
			prednisone	palliative therapy for metastatic castration resistant prostate cancer sing cabazitaxel and prednisone
		GUPDOC	dexamethasone	palliative therapy for metastatic hormone refractory prostate cancer
			docetaxel	palliative therapy for metastatic hormone refractory prostate cancer
			prednisone	palliative therapy for metastatic hormone refractory prostate cancer
		GUPDOCADT	docetaxel	first-line treatment of castration sensitive, metastatic prostate cancer using docetaxel and androgen deprivation therapy with LHRH agonist or LHRH antagonist with or without antiandrogen, or surgical castration
		GUPHDBIC	bicalutamide	Treatment of Prostate Cancer with High-Dose Bicalutamide
		GUPLHRH	Buserelin, Goserelin, leuprolide	therapy for prostate cancer using LHRH agonist
		GUPLHRHA	degarelix	therapy for prostate cancer using LHRH antagonist
		GUPMX	mitoxantrone	palliative therapy for hormone-refractory prostate cancer using mitoxantrone and prednisone
			prednisone	palliative therapy for hormone-refractory prostate cancer using mitoxantrone and prednisone

		GUPNSAA	bicalutamide	Non-Steroidal Treatment of Prostate Cancer, at 50 mg po daily
			flutamide	nonsteroidal treatment of prostate cancer
			nilutamide	prostate carcinoma patients who are intolerant to bicalutamide or flutamide, at 150 mg po daily
		GUPRAD	radium-223	therapy for metastatic castration resistant prostate cancer using radium- 223
		UGUMCSPABI	abiraterone	Therapy for Metastatic Castration-Sensitive Prostate Cancer using Abiraterone and Prednisone
			dexamethasone	Therapy for Metastatic Castration-Sensitive Prostate Cancer using Abiraterone and Prednisone
			prednisone	Therapy for Metastatic Castration-Sensitive Prostate Cancer using Abiraterone and Prednisone
		UGUMCSPAPA	apalutamide	Treatment of Metastatic Castration Sensitive Prostate Cancer using Apalutamide
		UGUMCSPENZ	enzalutamide	Therapy for Metastatic Castration Sensitive Prostate Cancer using Enzalutamide
		UGUNMPDAR	darolutamide	Treatment of Non-Metastatic Castration-Resistant Prostate Cancer using<br>Darolutamide
		UGUNMPENZ	enzalutamide	Therapy for Non-Metastatic Castration Resistant Prostate Cancer Using Enzalutamide

		UGUPABI	abiraterone	Palliative Therapy for Metastatic Castration Resistant Prostate Cancer Using Abiraterone and prednisone
			dexamethasone	Palliative Therapy for Metastatic Castration Resistant Prostate Cancer Using Abiraterone and prednisone
			prednisone	Palliative Therapy for Metastatic Castration Resistant Prostate Cancer Using Abiraterone and prednisone
		UGUPAPA	apalutamide	Treatment of Non- Metastatic Castration Resistant Prostate Cancer Using Apalutamide
		UGUPENZ	enzalutamide	Palliative Therapy for Metastatic Castration Resistant Prostate Cancer Using Enzalutamide
No treatment			No above treatments received in the first year after diagnosis	

[a] CCI for surgical treatments; Treatment type codes for radiation therapy; Protocol codes for ADT and chemotherapy;
[b] RP or other surgeries only or combination of RP/surgery and RT or ADT/chemo;
[c] RT only or combination with ADT/chemo;
[d] ADT/chemo only
NEC: not elsewhere classified; DAD: Discharge Abstract Database; BCCA: BC Cancer Agency

Table S2. Matched characteristics of patients with prostate cancer (cases) and controls at diagnosis (Cohort 1) and at 12 months before death (Cohort 2)

	Cohort 1			Cohort 2		
	Case (N=22672)	Control (N=45420)	P-value	Case (N=6942)	Control (N=13427)	P-value
**Age at diagnosis**			0.1599[1]			0.6731[1]
Mean (SD)	69.9 (8.94)	69.8 (8.99)		75.1 (9.54)	75.0 (9.47)	
Median (IQR)	69.0 (64.0, 76.0)	69.0 (63.0, 76.0)		75.0 (68.0, 82.0)	75.0 (68.0, 82.0)	
**Age at death**						0.8087[1]
Mean (SD)				81.2 (8.95)	81.2 (8.85)	
Median (IQR)				82.0 (75.0, 88.0)	82.0 (75.0, 88.0)	
**Elixhauser Comorbidity Index,** n (%)			0.7442[2]			0.1445[2]
0	18143 (80.0%)	36426 (80.2%)		3626 (52.2%)	7197 (53.6%)	
1	2000 (8.8%)	3949 (8.7%)		942 (13.6%)	1749 (13.0%)	
2	1074 (4.7%)	2086 (4.6%)		685 (9.9%)	1224 (9.1%)	
≥3	1455 (6.4%)	2959 (6.5%)		1689 (24.3%)	3257 (24.3%)	
**Health Authority,** n (%)			0.9919[2]			0.7830[2]
Interior	4544 (20.0%)	9117 (20.1%)		1637 (23.6%)	3149 (23.5%)	
Fraser	7091 (31.3%)	14256 (31.4%)		2080 (30.0%)	4076 (30.4%)	
Vancouver Coastal	4630 (20.4%)	9247 (20.4%)		1222 (17.6%)	2377 (17.7%)	
Vancouver Island	5206 (23.0%)	10429 (23.0%)		1632 (23.5%)	3170 (23.6%)	
Northern	1201 (5.3%)	2371 (5.2%)		370 (5.3%)	654 (4.9%)	
Missing				<5	<5	
**Health service delivery area,** n (%)			1.0000[2]			0.9949[2]
11 (East Kootenay)	295 (1.3%)	592 (1.3%)		130 (1.9%)	230 (1.7%)	
12 (Kootenay Boundary)	559 (2.5%)	1117 (2.5%)		164 (2.4%)	302 (2.2%)	

13 (Okanagan)	2226 (9.8%)	4478 (9.9%)		889 (12.8%)	1754 (13.1%)	
14 (Thompson Cariboo Shuswap)	1464 (6.5%)	2930 (6.5%)		454 (6.5%)	863 (6.4%)	
21 (Fraser East)	1353 (6.0%)	2720 (6.0%)		461 (6.6%)	882 (6.6%)	
22 (Fraser North)	2486 (11.0%)	4983 (11.0%)		693 (10.0%)	1367 (10.2%)	
23 (Fraser South)	3252 (14.3%)	6553 (14.4%)		926 (13.3%)	1827 (13.6%)	
31 (Richmond)	741 (3.3%)	1479 (3.3%)		166 (2.4%)	310 (2.3%)	
32 (Vancouver)	2198 (9.7%)	4388 (9.7%)		621 (8.9%)	1224 (9.1%)	
33 (North Shore – Coast Garibaldi))	1691 (7.5%)	3380 (7.4%)		435 (6.3%)	843 (6.3%)	
41 (South Vancouver Island)	2276 (10.0%)	4565 (10.1%)		775 (11.2%)	1515 (11.3%)	
42 (Central Vancouver Island)	2132 (9.4%)	4285 (9.4%)		643 (9.3%)	1261 (9.4%)	
43 (North Vancouver Island)	798 (3.5%)	1579 (3.5%)		214 (3.1%)	394 (2.9%)	
51 (Northwest)	326 (1.4%)	639 (1.4%)		106 (1.5%)	177 (1.3%)	
52 (Northern Interior)	621 (2.7%)	1229 (2.7%)		205 (3.0%)	378 (2.8%)	
53 (Northeast)	254 (1.1%)	503 (1.1%)		59 (0.8%)	99 (0.7%)	
99				<5	<5	
**Neighbourhood income quintile-QAIPPE,** n (%)			0.9800[2]			0.9613[2]
1	4243 (18.7%)	8539 (18.8%)		1612 (23.2%)	3147 (23.4%)	
2	4351 (19.2%)	8712 (19.2%)		1465 (21.1%)	2845 (21.2%)	
3	4308 (19.0%)	8622 (19.0%)		1314 (18.9%)	2546 (19.0%)	
4	4644 (20.5%)	9312 (20.5%)		1249 (18.0%)	2415 (18.0%)	
5	5064 (22.3%)	10126 (22.3%)		1240 (17.9%)	2370 (17.7%)	
9 unknown				62 (0.9%)	104 (0.8%)	
Missing	62 (0.3%)	109 (0.2%)				
**Follow-up in years**			0.4412[1]			
Mean (SD)	5.3 (2.55)	5.3 (2.56)				
Median (IQR)	5.2 (3.1, 7.5)	5.2 (3.1, 7.5)				
**PCa Stage,** n (%)						

Early	14427 (63.6%)
Advanced	5566 (24.6%)
Missing	2679 (11.8%)
**Tumor Grade,** n (%)	
Low grade	4946 (21.8%)
High grade	16069 (70.9%)
Missing	1657 (7.3%)
**Stage and Grade,** n (%)	
Early, low grade	3922 (17.3%)
Early, high grade	10006 (44.1%)
Advanced, low grade	183 (0.8%)
Advanced, high grade	4648 (20.5%)
Missing	3913 (17.3%)
**Primary Treatment,** n (%)	
RP	7118 (31.4%)
RT	6240 (27.5%)
ADT and/or chemo	3464 (15.3%)
WW or AS	5850 (25.8%)
**Early stage: Primary Treatment,** n (%)	
RP or other PCa-related surgeries	4085 (28.3%)
RT	4616 (32.0%)
ADT and/or chemotherapy	1260 (8.7%)
No treatment	4466 (31.0%)
**Advanced Stage: Primary Treatment,** n (%)	
RP or other PCa-related surgeries	2421 (43.5%)
RT	1468 (26.4%)
ADT and/or chemotherapy	1522 (27.3%)
No treatment	155 (2.8%)
**Low grade: Primary Treatment,** n (%)	

RP or other PCa-related surgeries	1019 (20.6%)
RT	377 (7.6%)
ADT and/or chemotherapy	109 (2.2%)
No treatment	3441 (69.6%)
**High grade: Primary Treatment,** n (%)	
RP or other PCa-related surgeries	5665 (35.3%)
RT	5618 (35.0%)
ADT and/or chemotherapy	2748 (17.1%)
No treatment	2038 (12.7%)

[1]Unequal variance two sample t-test; [2]Chi-Square p-value; SD: standard deviation; IQR: interquartile range (1st quartile, 3rd quartile); PCa: prostate cancer; RP: radical prostatectomy; RT: radiation therapy; ADT: androgen deprivation therapy; QAIPPE: quintile of annual income per person equivalent

Table S3. Duration of follow-up and costs per 100 days for cases and controls by interval

		Case[a]	Control[a]	Cost attributable to prostate cancer[b]
**Cohort 1**				
Interval I	N	22672	45420	
Follow-up in days	Mean (SD)	178.0 (15.3)	178.3 (14.4)	
	Median (IQR)	180 (180, 180)	180 (180, 180)	
	Min, Max	1, 180	1, 180	
Cost per 100 days	Mean (SD)	1442.3 (4261.66)	1046.2 (4066.69)	396.4 (334.6, 458.1)
	Median (IQR)	484.7 (245.5, 1059.3)	228.0 (52.5, 664.6)	
Interval II	N	22193	44542	
Follow-up in days	Mean (SD)	355.7 (47.0)	356.4 (44.7)	
	Median (IQR)	365 (365, 365)	365 (365, 365)	
	Min, Max	1, 365	1, 365	
Cost per 100 days	Mean (SD)	5002.7 (6899.80)	1075.4 (3432.05)	3,920.0 (3,827.4, 4,012.5)
	Median (IQR)	3866.0 (1973.9, 6156.5)	268.6 (84.4, 752.7)	
Interval III	N	21080	42429	
Follow-up in days	Mean (SD)	355.9 (46.7)	356.6 (44.5)	
	Median (IQR)	365 (365, 365)	365 (365, 365)	
	Min, Max	1, 365	1, 365	
Cost per 100 days	Mean (SD)	2134.0 (4467.09)	1124.4 (3636.64)	1,009.3 (941.6, 1,077.1)
	Median (IQR)	752.1 (245.2, 2303.1)	260.7 (76.3, 747.1)	
Interval IV	N	20056	40508	
Follow-up in days	Mean (SD)	1348.8 (858.7)	1343.1 (859.9)	
	Median (IQR)	1313.5 (566.0, 2103.0)	1306.0 (559.0, 2097.0)	
	Min, Max	1, 2920	1, 2920	
Cost per 100 days	Mean (SD)	2275.7 (10780.88)	1283.0 (3218.52)	992.3 (840.5, 1,144.1)
	Median (IQR)	787.1 (290.7, 2070.1)	412.3 (149.4, 1192.5)	
**Cohort 2**				
Interval V	N	6942	13427	
Follow-up in days	Mean (SD)	365 (0)	365 (0)	

	Median (IQR)	365 (365, 365)	365 (365, 365)	
	Min, Max	365, 365	365, 365	
Cost per 100 days	Mean (SD)	12184.2 (11087.02)	9453.4 (12791.33)	2,728.7 (2,394.2, 3,063.3)
	Median (IQR)	9674.7 (4546.0, 16500.1)	5724.8 (1832.0, 12067.6)	

[a] observed; [b] estimated from a generalized estimating equation (GEE) linear regression model of cost per 100 days on the binary variable of case-control group where the case-control matches were considered as clusters; Interval I: before diagnosis (6 months before diagnosis); Interval II: initial care (12 months after diagnosis based on Cohort 1); Interval III: post-initial care (12-24 months after diagnosis based on Cohort 1); Interval IV: continuing care (24 months after diagnosis till the earliest of 12 months before death, the last date of observation, or 2019/12/31 based on Cohort 1); Interval V: terminal care (12 months before death based on Cohort 2)

Table S4. Costs per 100 days for cases in Cohort 1 and controls by cancer stage, tumor grade and primary treatment

	Interval I		Interval II		Interval III		Interval IV	
	Case	Control	Case	Control	Case	Control	Case	Control
**By Stage**								
Early								
N	14427	28820	14298	28572	13917	27870	13515	27107
Mean (SD)	1338.8 (4100.57)	952.3 (3706.12)	4371.3 (5549.42)	986.1 (3223.57)	1723.4 (3421.69)	1059.8 (3215.50)	1665.1 (4251.99)	1234.1 (3030.44)
Median (IQR)	449.3 (236.2, 987.8)	213.9 (50.1, 622.3)	3636.6 (1723.3, 5770.6)	255.8 (81.9, 706.8)	583.2 (210.2, 1834.5)	253.1 (74.8, 717.2)	654.3 (255.9, 1692.0)	405.1 (149.4, 1166.6)
Advanced								
N	5566	11177	5309	10769	4785	9805	4337	8999
Mean (SD)	1530.8 (4588.77)	1155.5 (4439.94)	7018.4 (8571.83)	1217.4 (3774.05)	3344.4 (6346.49)	1206.8 (4539.20)	4208.9 (21622.34)	1342.3 (3729.55)
Median (IQR)	537.3 (262.7, 1136.5)	238.5 (48.6, 711.5)	5596.0 (3599.9, 7895.9)	269.9 (80.5, 800.2)	1699.1 (471.1, 4045.0)	247.0 (66.6, 752.9)	1306.2 (426.7, 3641.2)	375.4 (132.0, 1133.8)
**By Grade**								
Low grade								
N	4946	9886	4914	9829	4826	9641	4733	9461
Mean (SD)	1319.7 (4478.72)	843.4 (3340.86)	2716.6 (4606.68)	899.3 (3197.54)	1562.1 (3380.54)	987.3 (3181.16)	1407.5 (2558.09)	1112.0 (2590.91)
Median (IQR)	424.1 (221.4, 942.2)	186.7 (41.6, 560.2)	1529.8 (420.3, 3695.0)	223.9 (68.4, 625.3)	452.6 (180.4, 1578.8)	226.3 (63.7, 639.0)	609.3 (238.6, 1516.5)	368.2 (138.1, 1077.7)
High grade								
N	16069	32091	15845	31697	15116	30425	14409	29106
Mean (SD)	1332.3 (3934.08)	957.0 (3590.30)	5350.3 (5804.15)	1037.7 (3270.45)	2210.5 (4530.96)	1094.9 (3246.53)	2329.1 (7077.35)	1289.8 (3218.41)

Median (IQR)	478.5 (246.9, 1005.2)	225.1 (52.1, 643.1)	4379.1 (2768.2, 6464.6)	267.1 (85.7, 738.4)	876.5 (266.7, 2439.1)	263.1 (77.5, 749.4)	823.5 (305.5, 2171.0)	419.4 (150.8, 1208.0)
**By Stage and Grade**								
Early, Low								
N	3922	7843	3900	7799	3838	7664	3765	7530
Mean (SD)	1311.4 (4684.41)	819.4 (2908.08)	2661.7 (4845.29)	879.6 (3034.63)	1534.5 (3330.77)	985.2 (3280.67)	1396.2 (2611.64)	1088.0 (2597.41)
Median (IQR)	415.0 (220.7, 934.9)	184.3 (41.6, 556.1)	1453.2 (410.9, 3608.4)	217.5 (67.5, 608.3)	445.9 (175.4, 1570.9)	222.7 (61.8, 632.9)	589.7 (230.5, 1490.0)	351.4 (131.7, 1036.9)
Early, High								
N	10006	19972	9944	19840	9673	19382	9384	18828
Mean (SD)	1216.5 (3456.35)	904.9 (3407.63)	4746.6 (3925.55)	977.5 (3183.98)	1779.9 (3394.18)	1055.6 (3090.19)	1755.8 (4780.65)	1265.0 (3093.58)
Median (IQR)	444.9 (237.8, 945.9)	219.8 (52.5, 622.8)	4099.3 (2439.1, 6046.4)	263.0 (86.5, 712.8)	649.3 (226.4, 1905.4)	263.2 (78.6, 732.5)	673.2 (266.8, 1762.7)	423.9 (155.1, 1206.1)
Advanced, Low								
N	183	371	182	370	180	364	176	355
Mean (SD)	956.3 (2088.07)	676.0 (1786.31)	5304.5 (3444.27)	845.2 (2651.32)	2160.8 (3433.04)	854.7 (2165.84)	1265.7 (2486.58)	959.4 (1919.56)
Median (IQR)	448.2 (211.6, 747.5)	144.7 (24.0, 462.8)	4241.2 (3563.5, 6361.0)	200.6 (51.7, 531.9)	655.9 (183.3, 3246.0)	172.9 (34.4, 532.7)	500.1 (233.6, 1146.9)	290.2 (110.3, 921.9)
Advanced, High								
N	4648	9294	4526	9104	4173	8510	3852	7939

Mean (SD)	1408.2 (4627.86)	971.5 (3671.49)	6726.1 (7252.62)	1098.9 (3421.52)	3213.2 (6328.69)	1108.9 (3501.31)	3678.8 (11068.97)	1305.8 (3589.42)
Median (IQR)	514.6 (259.0, 1053.5)	220.4 (43.4, 636.3)	5647.5 (3665.0, 7818.4)	250.1 (76.3, 733.4)	1651.1 (456.4, 3957.2)	240.1 (65.3, 726.1)	1267.7 (417.9, 3369.5)	370.4 (131.2, 1114.7)
**By Primary Treatment**								
RP								
N	7118	14220	7062	14131	6937	13898	6813	13641
Mean (SD)	989.4 (2861.63)	798.0 (3576.16)	6085.3 (5741.38)	789.2 (2712.33)	1476.0 (2847.65)	868.8 (2953.80)	1412.7 (3524.41)	1046.2 (2879.12)
Median (IQR)	384.1 (212.7, 772.4)	154.4 (28.6, 468.7)	4808.3 (3717.5, 6697.0)	192.4 (60.5, 541.3)	426.0 (163.8, 1462.5)	188.5 (54.8, 560.8)	542.8 (221.5, 1416.2)	326.6 (122.3, 925.0)
RT								
N	6240	12443	6115	12260	5835	11775	5595	11328
Mean (SD)	1190.2 (3846.54)	965.1 (4051.89)	6182.2 (6641.05)	1007.8 (3038.07)	2111.5 (5252.31)	1121.8 (3665.97)	2368.5 (17916.14)	1318.2 (3191.65)
Median (IQR)	463.1 (246.5, 963.5)	231.9 (56.0, 659.8)	5460.0 (3311.5, 7268.9)	279.7 (92.5, 758.1)	946.9 (318.5, 2112.2)	280.0 (85.9, 766.6)	740.5 (308.5, 1949.5)	453.2 (165.4, 1247.9)
ADT and/or chemo								
N	3464	6884	3279	6570	2810	5747	2375	4961
Mean (SD)	2075.1 (4690.80)	1565.2 (4822.38)	4973.3 (9175.83)	1662.0 (4678.05)	4363.5 (6364.28)	1678.4 (5176.42)	5694.9 (12351.75)	1816.6 (4199.26)
Median (IQR)	755.0 (368.2, 1631.2)	397.0 (125.5, 1064.4)	2673.1 (1836.8, 4968.0)	451.0 (150.4, 1230.4)	2269.1 (1239.3, 5274.9)	429.0 (135.2, 1207.1)	2433.6 (1073.1, 6463.4)	607.4 (211.0, 1686.7)
No Treatment								

N	5850	11873	5737	11581	5498	11009	5273	10578
Mean (SD)	1887.3 (5555.99)	1127.3 (4125.58)	2429.6 (6239.80)	1163.3 (3719.74)	1848.7 (3625.29)	1160.7 (3369.47)	1752.4 (3206.26)	1300.5 (3097.76)
Median (IQR)	520.0 (251.7, 1257.0)	247.5 (62.3, 707.1)	777.8 (357.3, 2455.6)	288.4 (90.1, 806.2)	571.8 (222.4, 2053.5)	275.9 (82.2, 804.8)	789.9 (284.6, 1900.5)	434.2 (155.0, 1251.3)

Table S5. Estimated mean total costs attributable to prostate cancer six months prior diagnosis and per year after diagnosis by age, cancer stage, tumor grade and primary treatment in the first year

	6 Months Prior		Year 1		Year 2		Year 3+	
	**Mean**	**SE**	**Mean**	**SE**	**Mean**	**SE**	**Mean**	**SE**
**≥ 65 years old**								
Overall	745.3	71.8	14,552.6	221.5	3,850.8	152.9	3,879.2	233.0
By Grade								
Low	982.2	151.6	6,624.1	383.5	2,152.1	300.1	1,222.0	235.6
High	705.6	78.7	15,588.0	218.2	4,153.9	167.7	4,236.2	294.6
By Stage								
Early	719.1	83.8	12,632.2	223.4	2,623.1	160.2	1,939.4	202.3
Advanced	667.4	158.4	21,104.2	543.7	7,826.7	397.5	10,280.8	829.9
Primary treatment								
Radical prostatectomy and other related surgeries	259.9	119.4	20,429.1	402.0	2,419.5	223.2	1,711.4	280.6
Radiation therapy	399.6	126.6	19,452.3	377.8	3,357.7	261.9	2,944.9	476.2
Androgen deprivation therapy and/or chemotherapy	926.7	177.6	11,951.9	614.2	9,186.8	530.5	13,230.7	951.6
No treatment	1,456.1	155.5	5,277.4	383.7	2,393.2	266.7	1,849.0	245.7
Primary treatment for low grade								
Radical prostatectomy and other related surgeries	319.4	191.6	16,643.5	727.9	1,796.1	761.6	329.8	407.0
Radiation therapy	324.8	380.8	13,169.4	1,077.9	326.7	1,186.4	-238.6	1,060.3

Androgen deprivation therapy and/or chemotherapy	342.8	662.5	8,994.4	1,922.8	5,667.3	1,751.6	3,589.5	1,314.8
No treatment	1,200.2	192.8	3,937.6	457.0	2,272.5	344.9	1,456.8	279.2
Primary treatment for high grade								
Radical prostatectomy and other related surgeries	183.1	86.8	19,004.0	273.4	2,686.1	233.3	1,990.1	320.0
Radiation therapy	415.7	133.0	18,734.9	305.4	3,216.1	241.9	2,821.7	500.4
Androgen deprivation therapy and/or chemotherapy	981.9	195.8	11,775.2	709.5	9,612.7	562.0	12,960.1	1,088.5
No treatment	2,073.2	265.6	5,986.7	523.1	2,453.7	402.4	2,444.9	408.3
Primary treatment for early stage								
Radical prostatectomy and other related surgeries	236.0	184.0	19,978.2	629.7	1,130.5	293.9	737.1	357.4
Radiation therapy	213.0	107.7	16,202.3	227.7	1,931.2	245.7	1,012.1	247.9
Androgen deprivation therapy and/or chemotherapy	944.6	285.1	9,260.0	683.9	8,555.3	671.5	8,125.4	1,375.9
No treatment	1,533.4	170.2	4,767.9	387.7	2,432.1	285.4	1,874.1	257.8
Primary treatment for advanced stage								
Radical prostatectomy and other related surgeries	217.4	140.1	21,483.9	485.6	4,339.1	382.6	3,280.6	516.9
Radiation therapy	1,062.2	405.3	30,241.4	1,452.8	8,628.4	854.0	11,042.8	2,308.1

Androgen deprivation therapy and/or chemotherapy	1,015.8	281.6	13,807.6	911.2	12,241.8	939.3	23,624.2	1,911.0
No treatment	-987.9	861.5	8,490.0	2,651.7	7,526.7	2,217.4	2,503.4	3,032.4
**< 65 years old**								
Overall	635.6	85.4	13,723.7	245.9	3,295.6	222.3	3,054.2	750.0
By Grade								
Low	655.8	205.6	6,620.7	290.4	2,010.2	249.2	859.9	208.9
High	578.0	78.6	16,088.8	275.7	3,849.1	303.7	2,687.4	263.2
By Stage								
Early	632.5	113.5	11,670.5	264.2	1,966.6	174.3	765.5	146.4
Advanced	717.4	149.2	21,200.1	659.4	7,732.9	815.0	10,887.4	3,416.2
Primary treatment								
Radical prostatectomy and other related surgeries	443.1	85.1	17,970.6	259.8	1,979.0	195.8	901.2	189.6
Radiation therapy	418.3	120.6	16,780.6	485.5	4,447.2	829.0	6,828.1	3,509.7
Androgen deprivation therapy and/or chemotherapy	805.7	359.1	12,610.0	2,174.7	14,970.1	1,333.9	21,413.3	3,941.8
No treatment	1,172.8	274.2	2,837.3	498.0	2,811.5	293.9	1,169.3	252.1
Primary treatment for low grade								
Radical prostatectomy and other related surgeries	417.0	138.7	16,093.2	388.6	738.7	420.0	170.6	327.5
Radiation therapy	23.3	339.4	8,992.9	522.0	137.9	847.8	568.5	727.1
Androgen deprivation therapy and/or chemotherapy	-1,792.1	2,487.4	3,986.8	1,638.1	9,530.7	2,855.5	649.2	716.9
No treatment	895.4	324.8	1,631.9	352.0	2,785.9	328.1	1,247.8	286.0

Primary treatment for high grade								
Radical prostatectomy and other related surgeries	392.3	82.6	17,801.4	264.5	2,353.1	219.8	1,150.9	217.6
Radiation therapy	478.0	119.0	17,303.8	496.0	4,459.7	921.2	2,999.3	482.7
Androgen deprivation therapy and/or chemotherapy	1,025.6	366.9	12,213.2	2,340.4	16,327.3	1,389.3	19,679.6	3,011.5
No treatment	1,783.0	549.5	3,773.6	915.8	3,107.3	707.6	1,057.0	563.3
Primary treatment for early stage								
Radical prostatectomy and other related surgeries	430.9	114.2	16,707.2	326.2	993.4	224.5	99.2	203.1
Radiation therapy	263.1	113.3	13,424.7	399.0	1,584.3	356.5	1,178.4	316.8
Androgen deprivation therapy and/or chemotherapy	1,123.0	555.4	9,069.9	1,354.9	14,931.1	2,405.8	5,972.4	1,458.7
No treatment	1,198.6	328.0	2,929.6	594.7	2,847.1	333.2	1,088.8	269.6
Primary treatment for advanced stage								
Radical prostatectomy and other related surgeries	486.7	156.7	20,691.8	473.2	3,962.9	399.0	2,473.6	449.4
Radiation therapy	910.3	377.3	28,663.3	1,548.9	16,472.5	4,061.3	32,546.2	19,742.4
Androgen deprivation therapy and/or chemotherapy	959.2	456.7	14,829.8	3,365.0	15,864.7	1,806.9	32,028.2	6,465.8
No treatment	5,403.9	2,755.0	5,915.1	5,638.2	7,974.2	5,445.7	1,274.3	2,332.6

Estimated from a generalized estimating equation linear regression model on the binary variable of case-control group where the case-control matches were considered as clusters.

Table S6. Subgroup analysis: Characteristics of patients with prostate cancer at diagnosis (those with at least 5 years of follow-up) by age group

	Age < 65 (N=4045)	Age ≥ 65 (N=7686)
**Age at diagnosis**		
Mean (SD)	59.4 (3.70)	72.8 (5.82)
Median (IQR)	60.0 (57.0, 63.0)	72.0 (68.0, 77.0)
**Elixhauser Comorbidity Index, n (%)**		
0	3626 (89.6%)	6239 (81.2%)
1	243 (6.0%)	741 (9.6%)
2	96 (2.4%)	355 (4.6%)
⩾3	80 (2.0%)	351 (4.6%)
**PCa Stage,** n (%)		
Early	2755 (68.1%)	5237 (68.1%)
Advanced	766 (18.9%)	1386 (18.0%)
Missing	524 (13.0%)	1063 (13.8%)
**Tumor Grade,** n (%)		
Low grade	1271 (31.4%)	1651 (21.5%)
High grade	2641 (65.3%)	5747 (74.8%)
Missing	133 (3.3%)	288 (3.7%)
**Stage and Grade,** n (%)		
Early, low grade	965 (23.9%)	1214 (15.8%)
Early, high grade	1708 (42.2%)	3908 (50.8%)
Advanced, low grade	54 (1.3%)	50 (0.7%)
Advanced, high grade	693 (17.1%)	1257 (16.4%)
Missing	625 (15.5%)	1257 (16.4%)
**Primary Treatment,** n (%)		
RP or other PCa-related surgeries	2147 (53.1%)	2090 (27.2%)
RT	801 (19.8%)	2510 (32.7%)
ADT and/or chemotherapy	131 (3.2%)	990 (12.9%)
No treatment	966 (23.9%)	2096 (27.3%)
**Early stage: Primary Treatment,** n (%)		
RP or other PCa-related surgeries	1288 (46.8%)	1200 (22.9%)
RT	684 (24.8%)	2063 (39.4%)
ADT and/or chemotherapy	50 (1.8%)	472 (9.0%)
No treatment	733 (26.6%)	1502 (28.7%)
**Advanced Stage: Primary Treatment,** n (%)		
RP or other PCa-related surgeries	589 (76.9%)	681 (49.1%)
RT	102 (13.3%)	379 (27.3%)

ADT and/or chemotherapy	63 (8.2%)	277 (20.0%)
No treatment	12 (1.6%)	49 (3.5%)
**Low grade: Primary Treatment,** n (%)		
RP or other PCa-related surgeries	427 (33.6%)	272 (16.5%)
RT	119 (9.4%)	138 (8.4%)
ADT and/or chemotherapy	10 (0.8%)	53 (3.2%)
No treatment	715 (56.3%)	1188 (72.0%)
**High grade: Primary Treatment,** n (%)		
RP or other PCa-related surgeries	1631 (61.8%)	1720 (29.9%)
RT	679 (25.7%)	2348 (40.9%)
ADT and/or chemotherapy	116 (4.4%)	850 (14.8%)
No treatment	215 (8.1%)	829 (14.4%)

SD: standard deviation; IQR: interquartile range (1st quartile, 3rd quartile); PCa: prostate cancer; RP: radical prostatectomy; RT: radiation therapy; ADT: androgen deprivation therapy

Table S7. Sensitivity analysis: Comparison of patient characteristics based on two definition of tumor grade

	**Age < 65**		**Age >=65**	
	**Definition 1 (< 7 vs >=7)**	**Definition 2 (<=3+4 vs >=4+3)**	**Definition 1 (< 7 vs >=7)**	**Definition 2 (<=3+4 vs >=4+3)**
**Tumor Grade,** n (%)				
Low grade	1924 (29.5%)	3995 (61.3%)	3022 (18.7%)	6973 (43.2%)
High grade	4325 (66.4%)	2235 (34.3%)	11744 (72.7%)	7758 (48.0%)
Missing	269 (4.1%)	288 (4.4%)	1388 (8.6%)	1423 (8.8%)
**Stage and Grade,** n (%)				
Early, low grade	1536 (23.6%)	3069 (47.1%)	2386 (14.8%)	5434 (33.6%)
Early, high grade	2673 (41.0%)	1130 (17.3%)	7333 (45.4%)	4264 (26.4%)
Advanced, low grade	77 (1.2%)	433 (6.6%)	106 (0.7%)	630 (3.9%)
Advanced, high grade	1344 (20.6%)	981 (15.1%)	3304 (20.5%)	2769 (17.1%)
Missing	888 (13.6%)	905 (13.9%)	3025 (18.7%)	3057 (18.9%)
**Low grade: Primary Treatment,** n (%)				
RP or other PCa-related surgeries	585 (30.4%)	1829 (45.8%)	434 (14.4%)	1756 (25.2%)
RT	149 (7.7%)	647 (16.2%)	228 (7.5%)	1567 (22.5%)
ADT and/or chemotherapy	18 (0.9%)	65 (1.6%)	91 (3.0%)	394 (5.7%)
No treatment	1172 (60.9%)	1454 (36.4%)	2269 (75.1%)	3256 (46.7%)
**High grade: Primary Treatment,** n (%)				
RP or other PCa-related surgeries	2469 (57.1%)	1215 (54.4%)	3196 (27.2%)	1860 (24.0%)
RT	1192 (27.6%)	687 (30.7%)	4426 (37.7%)	3077 (39.7%)
ADT and/or chemotherapy	294 (6.8%)	246 (11.0%)	2454 (20.9%)	2148 (27.7%)
No treatment	370 (8.6%)	87 (3.9%)	1668 (14.2%)	673 (8.7%)

Table S8. Sensitivity analysis: Estimated mean total costs attributable to prostate cancer six months prior diagnosis and per year after diagnosis by age, tumor grade (Gleason ≤3+4: low grade and Gleason ≥4+3: high grade) and primary treatment in the first year

	**6 Months Prior**		**Year 1**		**Year 2**		**Year 3+**	
**≥ 65 years old**	**Mean**	**SE**	**Mean**	**SE**	**Mean**	**SE**	**Mean**	**SE**
By Grade								
Low	779.9	92.0	9,856.4	227.5	2,070.3	177.0	1,114.9	164.4
High	747.5	104.0	17,299.8	300.2	5,359.1	231.7	6,104.3	444.8
Primary treatment for low grade								
Radical prostatectomy and other related surgeries	287.9	123.0	17,308.8	332.5	1,581.5	310.5	419.8	279.6
Radiation therapy	160.3	152.9	13,348.1	422.0	778.5	328.9	393.3	356.6
Androgen deprivation therapy and/or chemotherapy	693.1	416.4	7,985.9	889.4	7,621.4	959.1	3,968.9	932.0
No treatment	1,354.4	162.0	4,328.3	361.1	2,321.3	276.8	1,547.2	243.8
Primary treatment for high grade								
Radical prostatectomy and other related surgeries	102.6	102.7	20,062.9	385.9	3,560.5	324.3	3,129.9	494.2
Radiation therapy	537.9	177.2	21,110.6	384.1	4,314.1	318.5	3,945.5	716.4
Androgen deprivation therapy and/or chemotherapy	1,023.5	211.5	12,385.4	801.0	9,881.2	627.3	14,580.7	1,261.0
No treatment	2,618.1	491.6	7,178.7	1,006.9	2,500.6	762.6	3,527.2	722.4
**< 65 years old**								

By Grade								
Low	593.7	119.6	10,053.8	211.8	1,860.6	167.0	731.3	139.7
High	614.3	93.5	18,714.7	453.5	5,879.9	551.9	4,683.0	484.3
Primary treatment for low grade								
Radical prostatectomy and other related surgeries	372.9	99.5	16,269.3	256.3	1,217.9	221.9	269.4	188.2
Radiation therapy	173.9	171.9	10,570.3	347.6	687.6	368.5	686.7	306.7
Androgen deprivation therapy and/or chemotherapy	199.9	883.3	8,234.4	1,784.4	12,111.6	2,237.7	3,890.4	1,647.5
No treatment	1,077.3	290.9	2,074.4	355.9	2,805.8	304.8	1,220.2	260.2
Primary treatment for high grade								
Radical prostatectomy and other related surgeries	435.4	100.9	19,285.3	411.6	3,312.9	356.7	2,034.2	372.9
Radiation therapy	652.5	146.9	21,828.4	759.4	7,198.6	1,602.0	4,672.3	815.5
Androgen deprivation therapy and/or chemotherapy	1,040.9	417.5	12,670.7	2,767.5	16,939.0	1,559.1	22,565.7	3,577.5
No treatment	1,628.3	999.7	3,303.5	1,558.2	3,840.6	1,611.1	918.2	1,297.9

Estimated from a generalized estimating equation linear regression model on the binary variable of case-control group where the case-control matches were considered as clusters.

Figure S1. Observed mean cost per 100 days for cases and controls in Cohort 1 by year

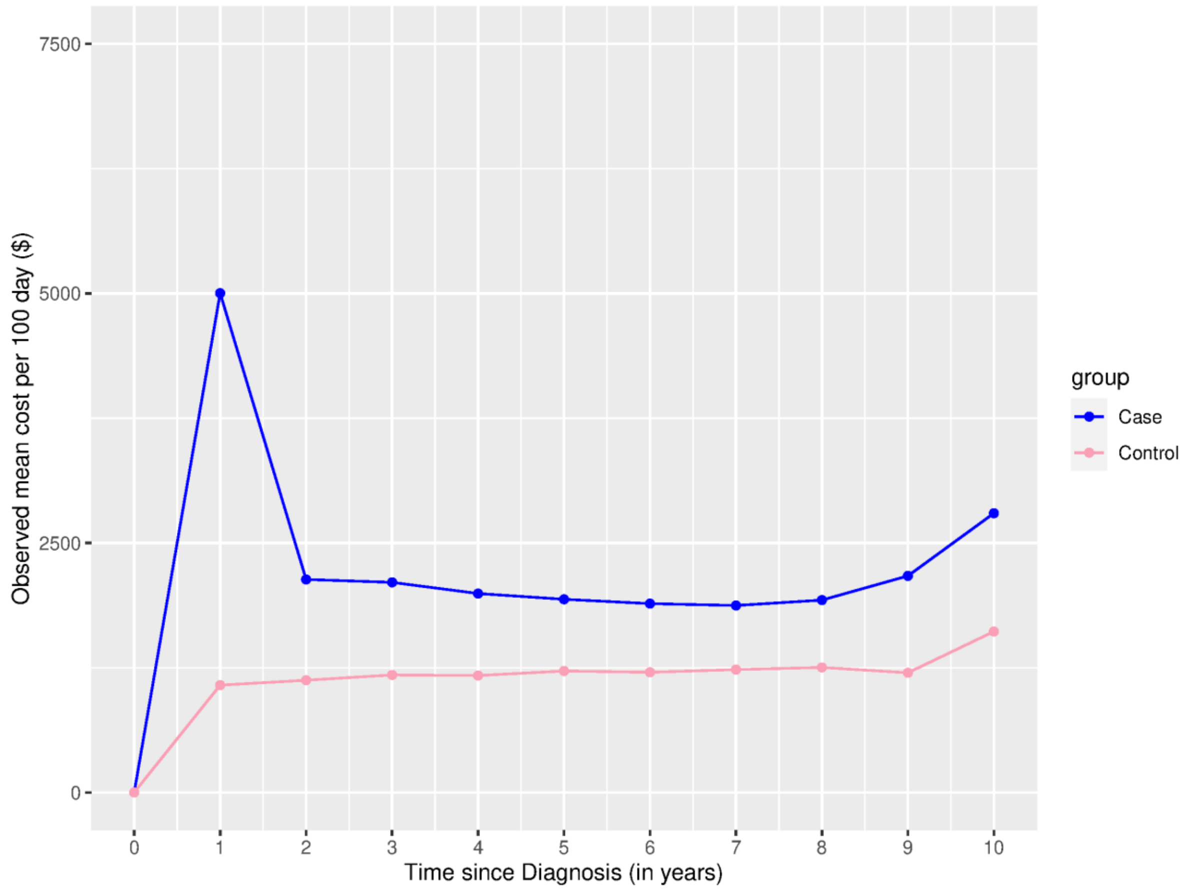

Figure S2. Estimated mean total costs in the first three years from diagnosis by age, cancer stage, tumor grade and primary treatment

< 65 years

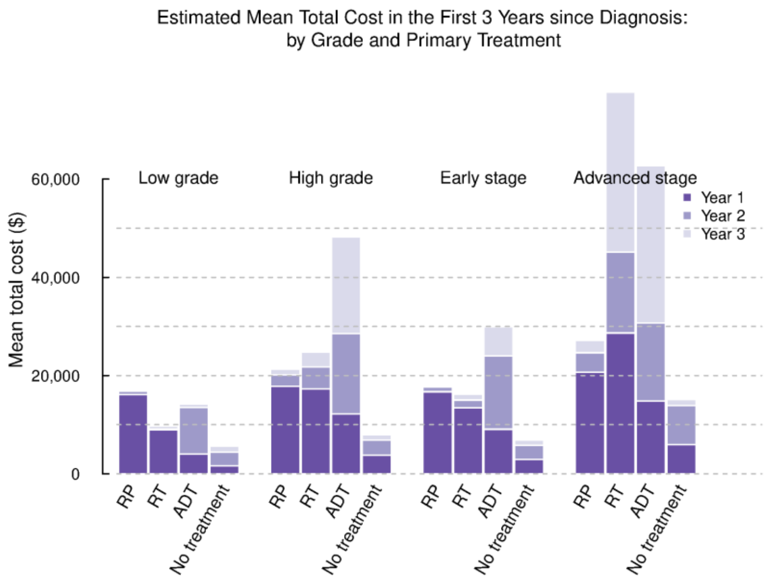

>= 65 years

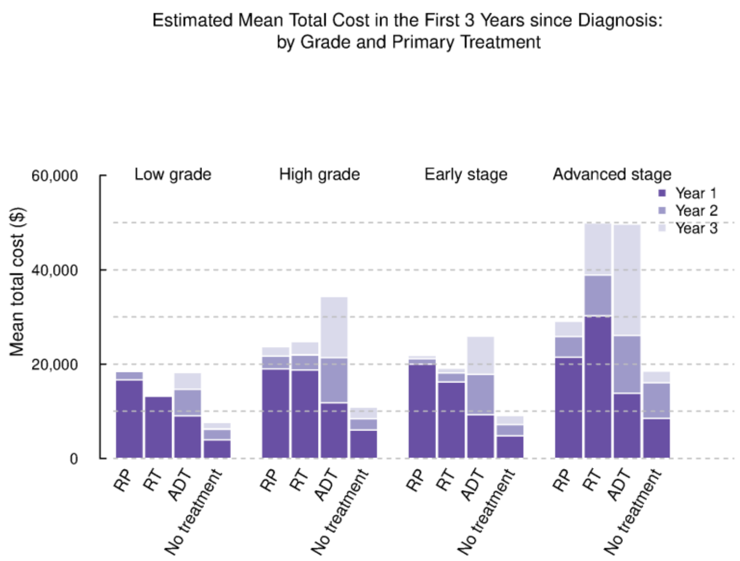

Figure S3. Subgroup analysis: Estimated mean total costs attributable to prostate cancer per year after diagnosis by age, cancer stage, tumor grade and primary treatment in the first year among those with at least 5 years of follow-up

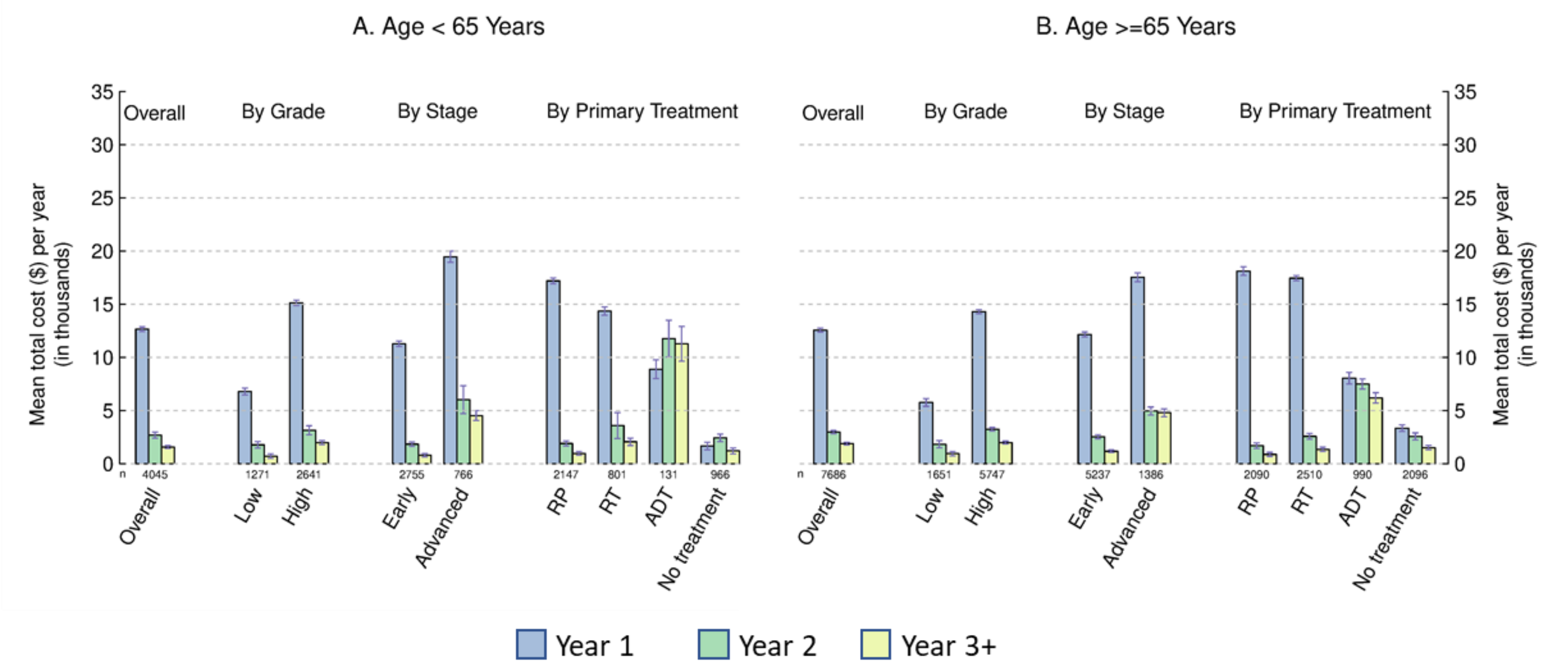

Year 1 or Interval II: initial care (12 months after diagnosis based on Cohort 1); Year 2 or Interval III: post-initial care (12-24 months after diagnosis based on Cohort 1); Year 3+ or Interval IV: continuing care (24 months after diagnosis till the earliest of 12 months before death, the last date of observation, or 2019/12/31 based on Cohort 1). Grey bars are standard errors. RP = radical prostatectomy or other PCa-related surgeries; RT = radiation therapy; ADT = androgen deprivation therapy and/or chemotherapy.

Figure S4. Sensitivity analysis: Estimated mean total costs attributable to prostate cancer per year after diagnosis by age, and two definitions of tumor grade

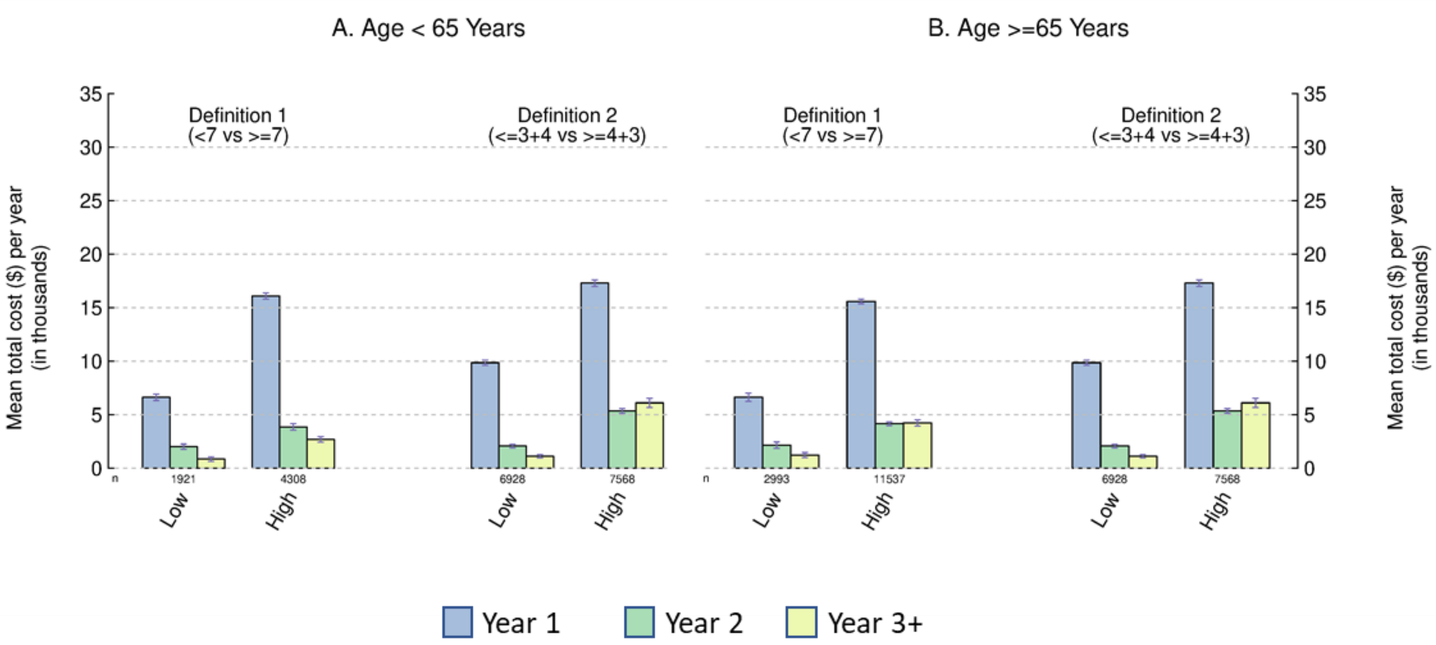

Year 1 or Interval II: initial care (12 months after diagnosis based on Cohort 1); Year 2 or Interval III: post-initial care (12-24 months after diagnosis based on Cohort 1); Year 3+ or Interval IV: continuing care (24 months after diagnosis till the earliest of 12 months before death, the last date of observation, or 2019/12/31 based on Cohort 1).

Figure S5. Sensitivity analysis: Estimated mean total costs attributable to prostate cancer per year after diagnosis by age, primary treatment in the first year and two definitions of tumor grade

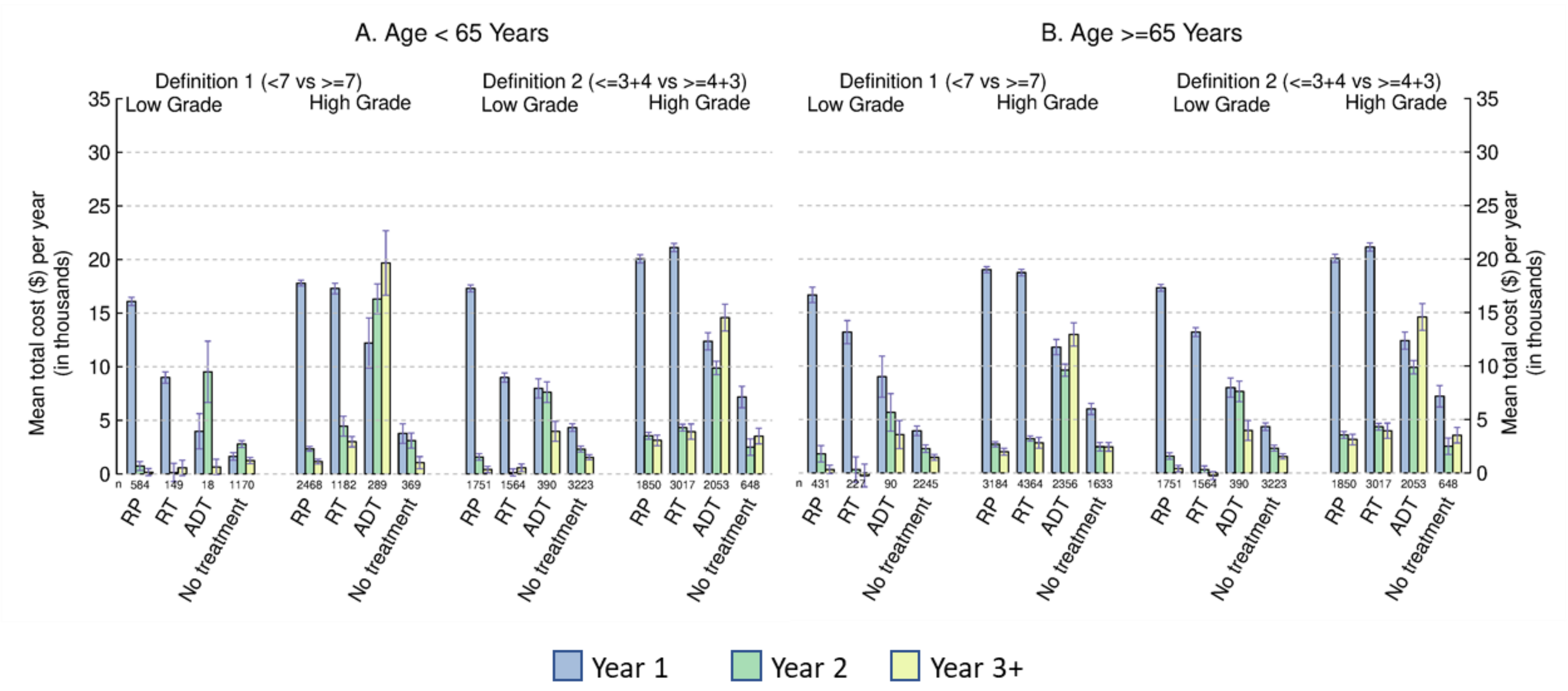

Year 1 or Interval II: initial care (12 months after diagnosis based on Cohort 1); Year 2 or Interval III: post-initial care (12-24 months after diagnosis based on Cohort 1); Year 3+ or Interval IV: continuing care (24 months after diagnosis till the earliest of 12 months before death, the last date of observation, or 2019/12/31 based on Cohort 1). Grey bars are standard errors. RP = radical prostatectomy or other PCa-related surgeries; RT = radiation therapy; ADT = androgen deprivation therapy and/or chemotherapy.
